# Using imputation to provide harmonized longitudinal measures of cognition across AIBL and ADNI

**DOI:** 10.1038/s41598-021-02827-6

**Published:** 2021-12-10

**Authors:** Rosita Shishegar, Timothy Cox, David Rolls, Pierrick Bourgeat, Vincent Doré, Fiona Lamb, Joanne Robertson, Simon M. Laws, Tenielle Porter, Jurgen Fripp, Duygu Tosun, Paul Maruff, Greg Savage, Christopher C. Rowe, Colin L. Masters, Michael W. Weiner, Victor L. Villemagne, Samantha C. Burnham

**Affiliations:** 1 The Australian e-Health Research Centre, CSIRO, Melbourne, Australia; 2 School of Psychological Sciences and Turner Institute for Brain and Mental Health, Monash University, Melbourne, Australia; 3 Department of Molecular Imaging and Therapy, Austin Health, Heidelberg, VIC Australia; 4 Florey Institute of Neuroscience and Mental Health, The University of Melbourne, Parkville, VIC Australia; 5 Centre for Precision Health, Edith Cowan University, Joondalup, WA Australia; 6 Collaborative Genomics and Translation Group, School of Medical and Health Sciences, Edith Cowan University, Joondalup, WA Australia; 7 School of Pharmacy and Biomedical Sciences, Faculty of Health Sciences, Curtin Health Innovation Research Institute, Curtin University, Bentley, WA Australia; 8 Department of Radiology and Biomedical Imaging, University of California-San Francisco, San Francisco, CA USA; 9 Cogstate Ltd., Melbourne, VIC Australia; 10 Department of Psychology, Macquarie University, Sydney, NSW Australia; 11 Department of Medicine, The University of Melbourne, Parkville, VIC 3052 Australia; 12 Department of Psychiatry, University of Pittsburgh School of Medicine, Pittsburgh, PA USA

**Keywords:** Alzheimer's disease, Computational biology and bioinformatics, Nanoscience and technology

## Abstract

To improve understanding of Alzheimer’s disease, large observational studies are needed to increase power for more nuanced analyses. Combining data across existing observational studies represents one solution. However, the disparity of such datasets makes this a non-trivial task. Here, a machine learning approach was applied to impute longitudinal neuropsychological test scores across two observational studies, namely the Australian Imaging, Biomarkers and Lifestyle Study (AIBL) and the Alzheimer's Disease Neuroimaging Initiative (ADNI) providing an overall harmonised dataset. MissForest, a machine learning algorithm, capitalises on the underlying structure and relationships of data to impute test scores not measured in one study aligning it to the other study. Results demonstrated that *simulated* missing values from one dataset could be accurately imputed, and that imputation of *actual* missing data in one dataset showed comparable discrimination (p < 0.001) for clinical classification to *measured* data in the other dataset. Further, the increased power of the overall harmonised dataset was demonstrated by observing a significant association between CVLT-II test scores (imputed for ADNI) with PET Amyloid-β in MCI *APOE*-ε4 homozygotes in the imputed data (N = 65) but not for the original AIBL dataset (N = 11). These results suggest that MissForest can provide a practical solution for data harmonization using imputation across studies to improve power for more nuanced analyses.

## Introduction

Aging populations are increasing the incidence of Alzheimer’s disease (AD) meaning that current health and economic frameworks will be overwhelmed without a cure or delay to the onset of disease. Thus, there exists an unprecedented challenge for understanding and preventing this disease. Alzheimer’s disease is a progressive, neurodegenerative disease characterized by neurodegeneration, synaptic loss, the accumulation of extracellular amyloid plaques and intracellular tau neurofibrillary tangles^1,2^. The major genetic risk factor for AD is the ε4 allele of apolipoprotein E (*APOE*)^3^, which has been consistently associated with cortical and subcortical grey matter atrophy and episodic memory decline^4^.

To increase understanding of the relationships between AD biomarkers and clinical disease progression, as well as the extent to which these relationships may be influenced by demographic, clinical or genetic factors, studies will require very large samples. This need will be increased if these factors are only present in small proportions of the population or if the magnitude of first or second interactions are small. One way to achieve additional power from existing data would be to combine data from existing studies such as the Australian Imaging, Biomarkers and Lifestyle (AIBL) Study of Ageing and Alzheimer’s Disease Neuroimaging Initiative (ADNI). In addition to increased statistical power, combining data across studies should also decrease bias due to sampling error, and improve the external validity of findings^5,6^. Harmonisation would further extend the utility of existing datasets^7^ and increase opportunities for multi-centre research collaborations^8–11^.

Despite these advantages, combining multiple existing datasets is not a common practice or trivial task due to differences in the study protocols. For example, different neuropsychological test batteries are administered for monitoring cognitive performance, measuring clinical disease progression and informing classification of clinical status (i.e., mild cognitive impairment (MCI) or dementia)^12,13^. While the different prospective AD studies largely converge in their neuropsychological test batteries on detailed measurement of memory, they differ in a number of ways including: (1) the number of other domains assessed, (2) the test paradigms used to measure specific domains (e.g. measurement of verbal or visual memory), (3) where the same test paradigm is used by both studies (e.g. list learning) it can be operationalized using different standardized tests (e.g. 15-word unstructured Rey Auditory Verbal Learning Test (RAVLT)^14^ in ADNI and the 16-word 5 trial T implicitly structured California Verbal Learning Test-Second edition (CVLT-II)^15^ in AIBL), (4) the number of scores derived from the different standardized tests and (5) their naming conventions.

In addition to variation in test scores, the design of AD prospective longitudinal studies is also different. For example, AIBL and ADNI use different retest intervals, have existed for different time periods and are composed of participants who have been evaluated from study inception as well as others that have joined later as part of multiple enrichment strategies.

In the context of data aggregation the term *harmonization* is defined as the process of transforming data from related outcomes to have similar response or scaling and thereby allow data from multiple studies to be integrated^16,17^. Statistical harmonization can be predominantly classified into three general methodologies^18^. First, different but related test scores can be combined across datasets by using a simple linear or z-transformation^16,19,20^. This approach of standardizing or normalizing scores generates data distributions that are unit-free thereby allowing outcomes from theoretically similar tests in different studies to be combined. Such methods require that the same underlying information is collected by similar tests and the strategy is not suitable for harmonising data with discrete values^21–24^, non-normal distributions or ceiling/floor effects^18^. Second, latent variable models can be utilised to determine underlying latent factors from a set of multiple test scores^21^. These models require common ‘anchor’ variables and assume that the measured test scores provide the same underlying information across studies that is captured by the latent construct^21^. Thirdly, imputation or maximum likelihood estimation can be used to impute values for a test not administered in one dataset that is administered in another. In this methodology test scores for the dataset that did not administer the test are considered missing in the joined dataset^25–27^ and imputation strategies are employed to impute the missing test scores^25–27^.

The present study proposes a new approach which uses a machine learning algorithm to impute and harmonize cognitive test scores across studies, as an extension to existing imputation methods of harmonization. The underlying structures and relationships of the measured data^21^ are exploited to impute cognitive test scores in one dataset where the test was not administered. This aligns that dataset to another dataset in which the test was measured, resulting in harmonized data across the two datasets. The estimation of the unmeasured test scores using an iterative imputation method based on Random forest^28^, called “MissForest”^29^ is proposed. Random forest is an advanced non-parametric machine learning algorithm which is able to handle mixed-type data (discrete, continuous and categorical variables) as well as data with a non-linear structure or that is non-normally distributed^30^. This investigation validates the proposed harmonization through imputation method using simulated missing values from two tests administered in both AIBL and ADNI datasets. Also, the extent to which the validity of the imputation holds is tested using different percentages of missing data. Further, the effectiveness of the method is demonstrated by imputing actual missing data for test scores only measured in one dataset. Finally, the utility of harmonised data to increase power in nuanced analyses is demonstrated by evaluating the relationship between cognition and PET Amyloid-β in *APOE* ε4-homozygotes with mild cognitive impairment (a small, very specific, sample of participants).

## Materials and methods

### Datasets

This study utilized neuropsychological, clinical, demographic and neuroimaging data collected as part of the AIBL and ADNI studies. The process of recruitment and enrolment in each of these studies has been described in detail elsewhere (ADNI^31^ and AIBL^32,33^ http://www.adni-info.org/index) and in the “Supplementary materials”.

Briefly, in AIBL, individuals classified clinically with mild cognitive impairment (MCI) or Alzheimer’s disease (AD) dementia were recruited from primary-care physicians or tertiary Memory Disorders Clinics at two study centres in Melbourne, Victoria and Perth, Western Australia. Cognitively normal (CN) older adults were recruited through advertisement or from spouses of participants in the study, at the same centres. The dataset contains data about neuroimaging, biomarkers, lifestyle, clinical information, and neuropsychological assessments. The follow-up data were collected every 18 months (18, 36, 54, 72 and 90 months)^31^. Note not all participants completed all the evaluations. Written informed consent was obtained from all participants. Approval for the study was obtained from the human research ethics committees of Austin Health, St Vincent’s Hospital, Edith Cowan University, and Hollywood Private Hospital. All methods were carried out in accordance with relevant guidelines and regulations and all participants or legal guardian(s)/legally authorized representatives gave their informed consent (for more details, see Ellis et al.^31^, https://aibl.csiro.au).

ADNI is a multi-centre longitudinal neuroimaging study, launched by the National Institute on Aging, the National Institute of Biomedical Imaging and Bioengineering, the Food and Drug Administration, private pharmaceutical companies and non-profit organizations in 2004. The dataset includes data from neuroimaging, biomarkers, clinical information, and neuropsychological assessments, as previously described^32,33^. The ADNI participants were followed prospectively, with follow-up time points at 3 months, 6 months, then every 6 months until up to 156 months. Note not all participants completed all the evaluations. A committee on human research at each participating institution approved the study protocol, and all participants or legal guardian(s)/legally authorized representatives gave their informed consent. All methods were carried out in accordance with relevant guidelines and regulations.

### Inclusion and exclusion criteria

In this study, clinical measures and neuropsychological tests that were common across AIBL and ADNI and that had less than 50% missing data in each of the respective datasets were included. For example, whilst Digit Span and Digit Symbol-Coding were measured in both datasets, more than 50% of the observations were missing in one of the original datasets, therefore they were excluded. To demonstrate the utility of the method, two tests not common across the datasets were also included, namely: the RAVLT from ADNI and the CVLT-II from AIBL. All included clinical and cognitive test scores, alongside their variable names in ADNI and AIBL datasets and their percentage of missingness are listed in Table S1.

We excluded a participant’s measurements at a specific time point if the clinical classification was missing and the classification could not be determined using the clinical classification of the participant at an adjacent timepoint. Further, a participant’s measurements at a specific time point were excluded if there were less than three completed neuropsychological test measurements for that time point.

This study included 1805 AIBL participants (CN = 1180, MCI = 297 and AD = 328), aged 72.42 ± 7.64 years with 777 males at baseline, and 2122 ADNI participants (CN = 791, MCI = 962, AD = 369), aged 73.32 ± 7.21 years with 1129 males at baseline.

### Joined dataset

A joined dataset was formed combining the AIBL and ADNI datasets in long format, where each row represented a single time point per subject. All missing data were coded as “NA”, this including data missing at random as well as systematically missing data (e.g. from the CVLT-II and RAVLT)^18^.

Cognitive tests used to provide a joined dataset across AIBL and ADNI included subtests from: California Verbal Learning Test-Second edition (CVLT-II)^15^, Rey Auditory Verbal Learning Test (RAVLT)^14^, and Logical Memory (LM) I and II (WMS; Story A only), 30-item Boston Naming Test (BNT)^34^, Digit Span and Digit Symbol-Coding subtests of the Wechsler Adult Intelligence Scale-Third edition (WAIS-III)^35^.

Time-dependent variables were defined as rate of change for each of the clinical and cognitive tests, calculated as the difference in test scores between the current and previous time point divided by the time elapsed between those two time points. Given that in the long format longitudinal data at each row is a single time point per subject, the time-dependent variables represent a longitudinal feature of each test score for a specific subject. These variables were included as predictors in the models. Time was calculated as the number of months since baseline.

The final joined dataset included 39 columns. Variables incorporated in the joined dataset included: (a) identifiers: participant ID and dataset ID; (b) demographic measurements: age, sex, years of education, clinical classification (CN, MCI and AD), and a genetic risk factor (carriage of the *APOE*-ɛ4 allele^3^); (c) clinical tests: Clinical Dementia Rating (CDR), and Mini-Mental State Examination (MMSE)^36^; (d) 14 cognitive test scores; and (e) time-dependent variables calculated for clinical tests and cognitive scores.

### Data preparation

Due to differences in naming conventions between ADNI and AIBL it was necessary to adopt a single variable name for the joined dataset. Table S1 lists the original name of the study variables from AIBL and ADNI, the description of the test scores and the names used for the variables in the joined dataset.

Further, data included in the joined dataset were modified to ensure the same representation and data type across the studies; this included: (1) converting the continuous values of the years of education in ADNI to ordinal values with four intervals (< 9, 9–12, 13–15, 15+) as in AIBL, (2) converting genetic risk factors data which are presented with characters in AIBL into categorical values as in ADNI, (3) converting gender in AIBL from data type characters to categorical values. The age for AIBL dataset was also calculated using date of birth and the date cognitive tests were administered.

### PET Aβ-amyloid for the case study

A subset of AIBL subjects (N = 1042) underwent Aβ-amyloid positron emission tomography (PET) imaging using either ^11^C-Pittsburgh compound-B (^11^C-PiB), ^18^F-NAV4694 (NAV), ^18^F-florbetaben (FBB), ^18^F-florbetapir (FBP) or ^18^F-flutemetamol (FLUTE). A subset of ADNI subjects (N = 1565) underwent Aβ-amyloid PET studies with either FBP or FBB. The AIBL PET images were smoothed to a uniform 8 mm full width half-maximum point spread function to match the PET pre-processing done in ADNI^37^. All PET images were spatially normalized with SPM8, using the prescribed Centiloid pipeline^38^. Then a tissue ratio, termed SUV ratio (SUVR), was computed using the ratio of the PET retention computed inside the neocortical Centiloid mask and the whole cerebellum. The SUVR was then transformed into Centiloids using each tracer’s respective linear transform^39^. Smaller subsets of the data from AIBL (33 observations from N = 11 subjects) and ADNI (108 observations from N = 54 subjects) including *APOE-*ε4/ε4 individuals with MCI were used to investigate the influence of the levels of Aβ-amyloid on memory performance.

### Statistical analysis and process of imputation

Baseline differences in demographic variables and months of follow-up between AIBL and ADNI datasets were analysed using a t-test for continuous variables with normal distribution, Mann–Whitney U-test for continuous variables with non-normal distribution, and χ^2^ for categorical variables.

After joining the AIBL and ADNI datasets together, all missing data coded as ‘NA’, which included test scores not measured for one dataset (systematic missing) and data missing at random, were imputed using MissForest^40^, a non-parametric, iterative imputation method based on Random forests^28^. For further details on the MissForest approach are provided by Stekhoven et al.^40^. Our model parameters included the 39 variables described in the previous section except for the participant ID and dataset ID (AIBL, ADNI). Note that the dataset ID was used later in validation steps but was not shown to the prediction models.

Using notation similar to Stekhoven et al.^40^, let *X* = *(X*_*1*_*, X*_*2*_, …, *X*_*p*_*)* to be a *r* × *p*-dimensional joint AIBL-ADNI dataset. For each outcome variable X_k_, i_obs_ indicates the indices of the subjects with observed values and i_mis_ indicates the indices of the subjects with missing values. y_obs_ are the observed values of X_k_, and y_mis_ are the missing values of variable X_k_. X_obs_ indicates a data matrix including the subjects with i_obs_ indices and all the variables except for X_k_. X_mis_ is a data matrix including the subjects with i_mis_ indices and all the variables except for X_k_.

For instance, to estimate CVLT-II test scores, y_obs_ represent the observed values of a CVLT-II score from AIBL, and y_mis_ represents values that are randomly missing from AIBL as well as the completely missing values from ADNI. MissForest required an initial value for missing data, here the median of the measured data in the joined dataset was used for continuous as well as discrete variables and for categorical variables the category with the highest frequency was used. MissForest then fit the random forest model with response y_obs_ and predictors X_obs_ (including the rest of neuropsychological test scores as well as clinical and demographic measures) to extract the underlying relationships between CVLT-II and the other observed data in X_obs_. Using this final, trained MissForest model and the other observed data in X_mis_, the missing values of CVLT (y_mis_) were imputed. The imputed data were then defined as the initial estimates and the procedure was repeated until the difference between the input and output estimates was sufficiently small (stopping criterion^40^).

We used the MissForest^40^ package in R^29^, which is an implementation of the Random Forest algorithm^28^ that iteratively creates non-parametric imputations of each variable. To have high precision the number of trees was set to the default value of 100. The number of iterations was chosen to be 10 if the stopping criterion was not already met. However, it has been shown that the algorithm normally reaches the stopping criterion in five iterations^40^. Since the neuropsychological test scores LMII and CVLT-II are integer values, after the imputation the estimated scores were rounded to the nearest integer.

### Method validation by simulating missing data

To evaluate the efficacy of the method and validate its utility, LMII and MMSE tests measured across both AIBL and ADNI were selected as outcome variables where a proportion of the data was simulated as missing. Here, let *n* and *m* be number of subjects with an observed outcome variable (e.g. LMII) in AIBL and ADNI datasets, respectively. Given the larger size of observations in ADNI compared to AIBL (*m* ~ 1.7*n), a subsample of ADNI with the same size as AIBL, herein called ADNI_sub_, was used for the validation steps to ensure comparable results across the evaluations. Also, in order to simplify the validation, the CVLT-II and RAVLT scores, only observed in one dataset, were excluded from these validation steps. The following two validation approaches were undertaken:

First validation approach: to provide an understanding of the impact of the different underlying structures and relationships in AIBL and ADNI, the differences between estimating missing data using observed data from the same dataset (i.e. ADNI data predicting missing ADNI data) as well as a different dataset (i.e. AIBL data predicting missing ADNI data), which represents the proposed harmonization approach were evaluated. The LMII (X_k,_ k = LMII) test score was chosen as the outcome variable and missing values for this test score were simulated. The accuracy of two models was compared: model 1 estimated the missing data based on the observed part of the same dataset; model 2 estimated the missing data using a secondary dataset which was joined with the first dataset. This validation step was also repeated where MMSE represented the outcome variable (X_k,_ k = MMSE).

In this validation approach the test dataset was selected from ADNI _sub_, which was randomly split into a 25% test (missing data) and 75% training (observed data) set used for model 1 (*i*_*obs1*_ $\subseteq ADNI$). A second training dataset representing a randomly assigned 75% subsample of the AIBL dataset was generated for model 2 (*i*_*obs2*_ $\subseteq AIBL$). As mentioned above, the size of training and test datasets across models 1 and 2 were the same to ensure fair comparisons between the models. The outcome variable (LMII or MMSE) in the test dataset (*i*_*mis*_ $\subseteq ADNI$) was simulated as missing; note the same test samples were used for both models. MissForest was applied to derive estimates of the missing values. The performance of the data imputation was examined on the test dataset using the mean absolute error (MAE) and the Pearson correlations between the estimated and the actual scores.

The performance metric, MAE, was calculated as:

1 $$MAE = \frac{1}{N}\sum\limits_{{i = 1}}^{N} {\left| {x_{i}^{{actual}} - x_{i}^{{imp}} } \right|}$$

where N is the number observation in the test dataset, ${x}_{i}^{imp}$ shows the imputed values, and ${x}_{i}^{actual}$ the actual value of the score measured for an observation point $i$ in the test dataset. To measure the variability the analyses were replicated 100 times. For each replication new ADNI_sub_ test and training data sets were randomly generated.

In order to determine if AIBL and ADNI had comparable accuracy at imputing missing data in each other, the validation was repeated with the test dataset and model 1 training data drawn from AIBL, and with model 2 training data drawn from ADNI.

Second validation approach: to test the limit of efficacy of the proposed method, the impact of varying degrees of missingness in the test dataset and different sizes of training datasets were evaluated. ADNI_sub_ was used as the training dataset and the test dataset was set as a randomly selected subset of AIBL equal to 10%, 20%, 30%, 40%, 50%, 60%, 70%, 80%, 90%, and 100% of the AIBL dataset and the outcome variable (LMII or MMSE) was simulated as missing. To provide variance estimates, this process was replicated 100 times. These analyses were also repeated for smaller training sets equal to 10% and 50% of participants in ADNI_sub_ (randomly selected).

### Data harmonization application

Unmeasured test scores (CVLT-II in ADNI and RAVLT in AIBL) were imputed using MissForest. The utility of the resulting imputed data was examined by comparing the distribution of the imputed and actual test scores through boxplots stratified by clinical classification (CN, MCI, AD). Further, the difference between the clinical classifications of both imputed and actual data was evaluated using Student’s t-tests and Cohen’s *d* effect sizes. Here, imputed ADNI CVLT-II scores were compared to the observed AIBL CVLT-II scores and imputed AIBL RAVLT scores were compared to the observed ADNI RAVLT scores. We also performed sensitivity analysis on CVLT-II and RAVLT scores using their original values in AIBL and ADNI (Details of this sensitivity analysis are given in the “Supplementary materials”).

### Data harmonization utility case study

The utility of harmonised data to increase power in nuanced analyses was evaluated by considering the influence of Aβ-amyloid levels on memory performance in *APOE* ε4-homozygotes with mild cognitive impairment (a small, specific sample of participants). The associations between Aβ-amyloid levels and the CVLT-II and RAVLT total immediate recall memory scores in the sample were evaluated for both the actual and the harmonized datasets using regression analyses.

## Results

Significant differences in distribution of clinical classification, sex, *APOE* ε4 status, level of education as well as age between AIBL and ADNI were observed, Table 1. Note that due to participant drop-out and enrichment strategies different participants completed different numbers of evaluations^31–33^.

**Table 1:** Demographics table for AIBL, ADNI and the joined dataset.

	AIBL dataset (N = 1791)	ADNI dataset (N = 2122)	Joined harmonised dataset (N = 3913)	Statistic (df)	p-value
Clinical Classification CN/MCI/AD [N (%)]	1179 (65.83)/296 (16.53)/316 (17.64)	791 (36.95)/962 (41.38)/369 (21.68)	1970 (50.35)/1258 (32.15)/685 (17.51)	χ^2^(2) = 408.03	< 0.001
Sex:female [N (%)]	1015 (56.67)	993 (46.80)	2008 (51.32)	χ^2^(1) = 37.53	< 0.001
Years of age at baseline [mean (sd)]	72.42 (7.64)	74.10 (7.26)	72.91 (7.42)	t(3719) = − 3.75	< 0.001
*APOE*-ε4 allele 0/1/2 [N (%)]	935 (52.21)/449 (25.07)/90 (5.030)	1114 (52.50)/739 (34.83)/194 (9.14)	2049 (52.36)/1188 (30.36)/284 (7.26)	χ^2^(2) = 32.11	< 0.001
Months of follow-up [mean (sd)]	39.98 (35.77)	37.54 (35.72)	38.66 (35.76)	W = 1,961,934	0.077
Years of education < 9/9–12/13–15/15+ [N (%)]	185 (10.33)/656 (36.63)/345 (19.26)/549 (30.65)	23 (1.08)/288 (13.57)/401 (18.90)/1410 (66.45)	208 (5.32)/944 (24.12)/746 (19.06)/1959 (50.06)	χ^2^(3) = 619.66	< 0.001

p-values present statistical differences between AIBL and ADNI participants calculate with two-sample t-test for continuous data with normal distribution, Mann–Whitney U test for continuous data with non-normal distribution, and χ^2^ testing for categorical variables).

### Validation results

The mean absolute errors (MAE; mean ± standard error) of the first validation approach indicated that the imputed ADNI LMII (range 0–25) and ADNI MMSE (range 0–30) scores estimated using the observed part of the ADNI dataset were 1.95 ± 0.00 and 1.60 ± 0.00, respectively. The correlation (mean ± standard error) between the imputed values for ADNI LMII and ADNI MMSE scores estimated using the observed part of the ADNI dataset were 0.91 ± 0.00 and 0.81 ± 0.00, respectively. The MAE (mean ± standard error) for the imputed ADNI LMII and ADNI MMSE scores estimated using the AIBL dataset were 2.40 ± 0.00 and 2.19 ± 0.00, respectively. Also, the correlation between ADNI LMII and ADNI MMSE scores estimated using the AIBL dataset were 0.88 ± 0.00 and 0.76 ± 0.00, respectively.

Further, MAE (mean ± standard error) for the imputed AIBL LMII and AIBL MMSE scores using the observed part of the AIBL dataset, were 1.64 ± 0.00 and 1.31 ± 0.00, respectively. The correlation (mean ± standard error) between the imputed AIBL LMII and AIBL MMSE scores using the observed part of the AIBL dataset were 0.92 ± 0.00 and 0.88 ± 0.00. The MAE (mean ± standard error) for the estimated AIBL LMII and AIBL MMSE scores using the ADNI dataset, were 1.65 ± 0.00 and 1.44 ± 0.01, respectively. The correlation (mean ± standard error) between the imputed AIBL LMII and AIBL MMSE scores using the ADNI dataset, were 0.92 ± 0.00 and 0.86 ± 0.00.

**Figure 1:**
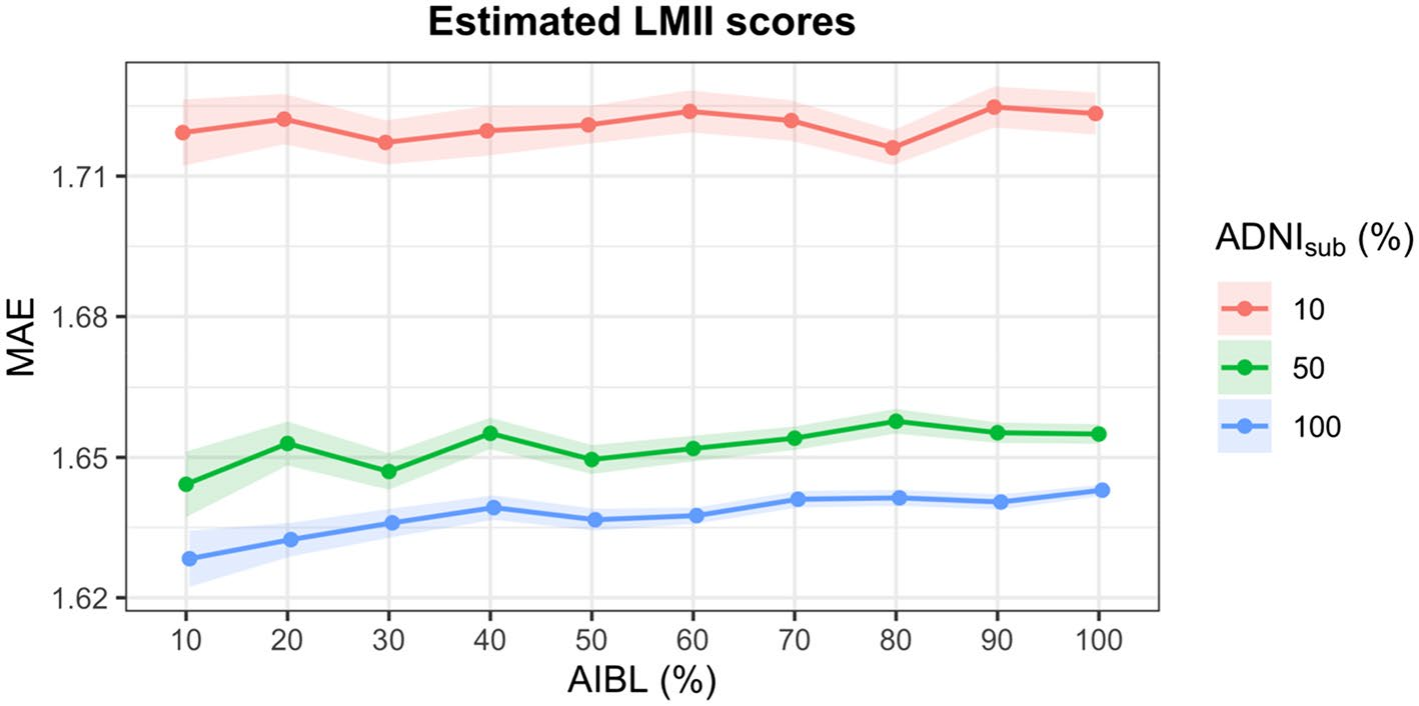
Performance of imputed simulated missing AIBL LMII scores with different sizes of training and missing data: the performance is calculated using the mean absolute error (MAE) of imputed and actual data. Different size of training data samples of the ADNI dataset (equal to the size of 10%, 50%, and 100% of the AIBL dataset) and different sizes of simulated missing data samples of 10%, 20%, 30%, 40%, 50%, 60%, 70%, 80%, 90%, and 100% of the AIBL dataset were used. The results show high prediction accuracy even for training (reference) dataset with ten times smaller sample size compared to the size of the joining dataset.

**Figure 2:**
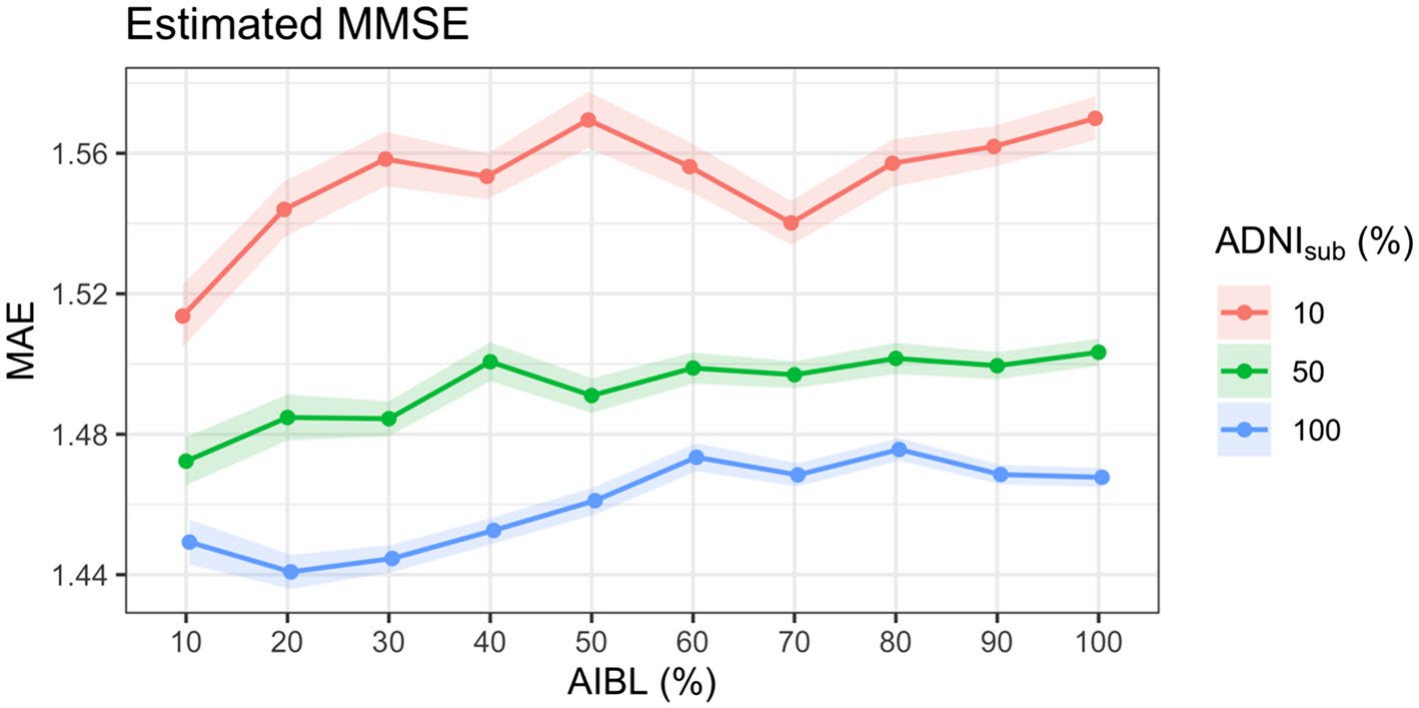
Performance of imputed simulated missing AIBL MMSE scores with different sizes of training and missing data: the performance is calculated using the mean absolute error (MAE) of imputed and actual data. Different size of training data samples of the ADNI dataset (equal to the size of 10%, 50%, and 100% of the AIBL dataset) and different sizes of simulated missing data samples of 10%, 20%, 30%, 40%, 50%, 60%, 70%, 80%, 90%, and 100% of the AIBL dataset were used. The results show high prediction accuracy even for a training dataset with ten times smaller sample size compared to the size of the joining data.

**Figure 3:**
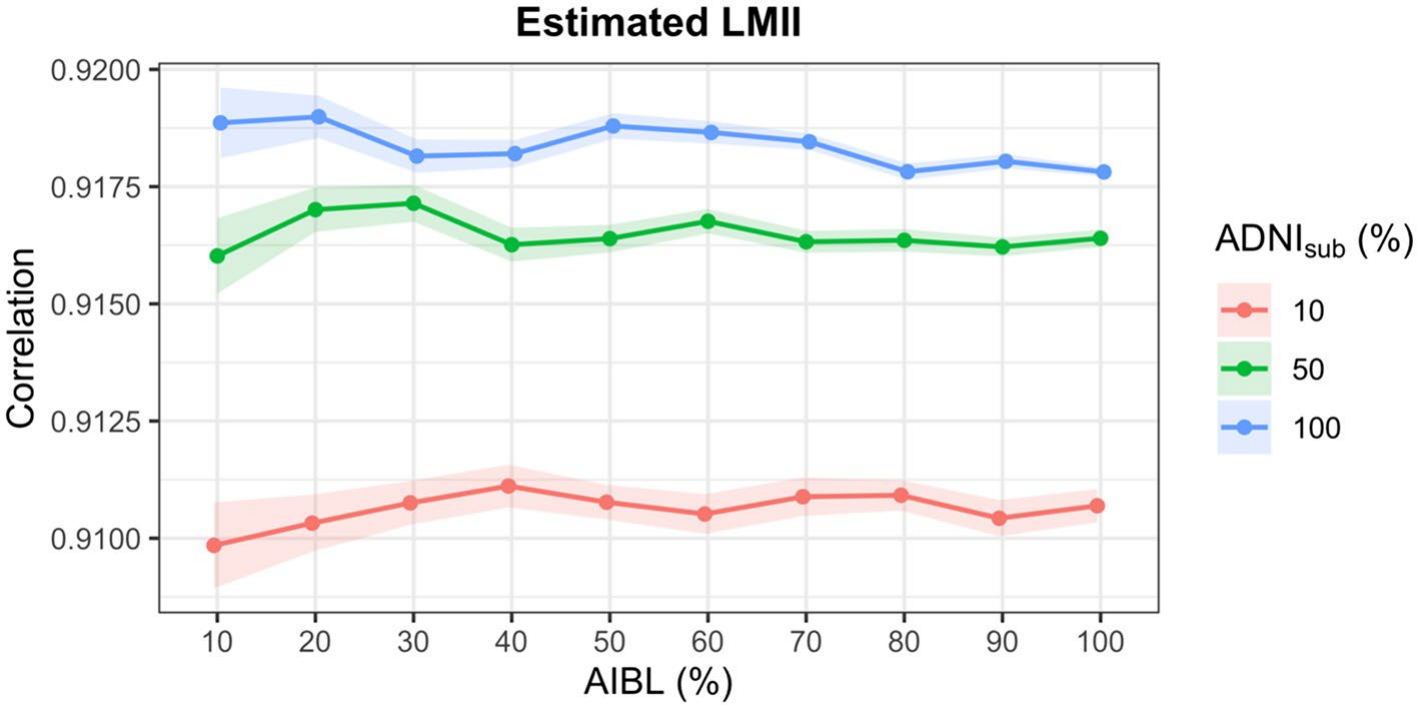
Performance of imputed simulated missing AIBL LMII scores with different sizes of training and missing data: the performance is calculated using the correlation between the imputed and actual data. Different size of training data samples of the ADNI dataset (equal to the size of 10%, 50%, and 100% of the AIBL dataset) and different sizes of simulated missing data samples of 10%, 20%, 30%, 40%, 50%, 60%, 70%, 80%, 90%, and 100% of the AIBL dataset were used. The results show high prediction accuracy even for training (reference) dataset with 10 times smaller sample size compared to the size of the joining dataset.

**Figure 4:**
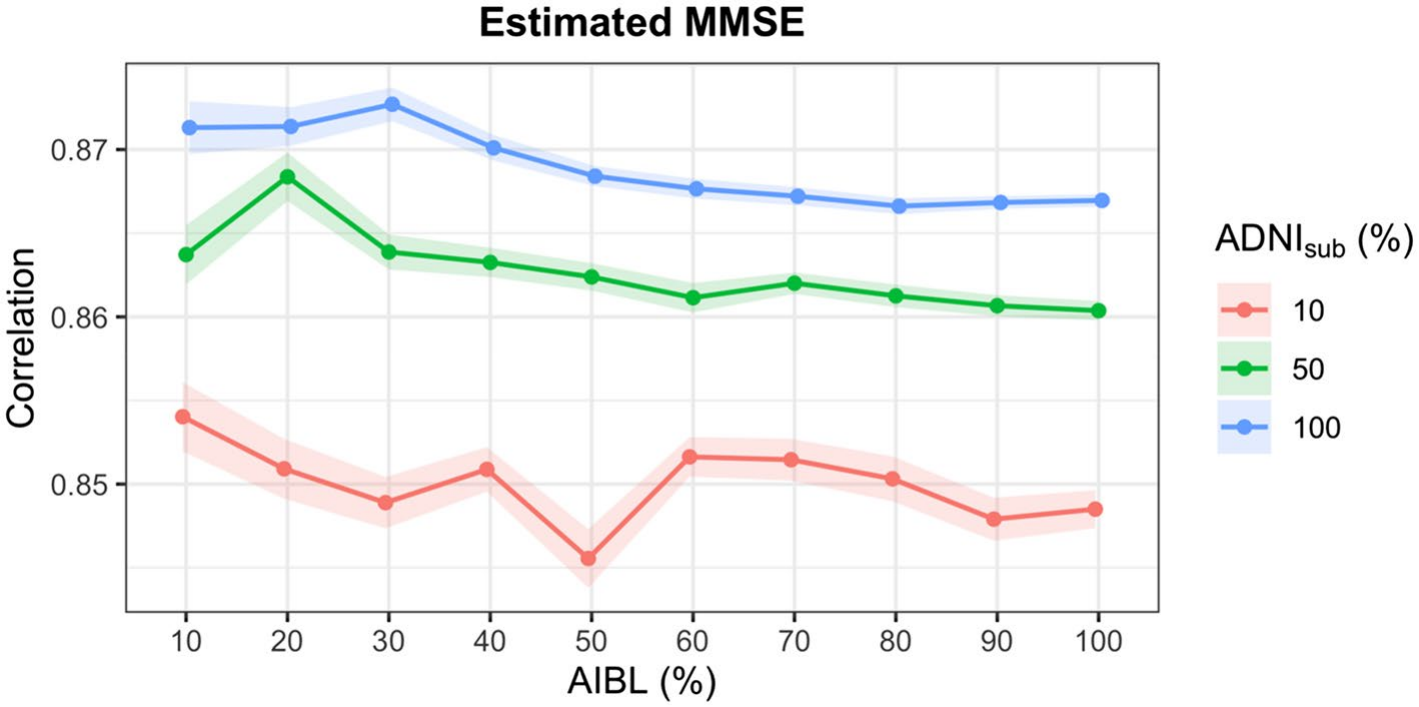
Performance of imputed simulated missing AIBL MMSE scores with different sizes of training and missing data: The performance is calculated using the correlation between the imputed and actual data. Different size of training data samples of the ADNI dataset (equal to the size of 10%, 50%, and 100% of the AIBL dataset) and different sizes of simulated missing data samples of 10%, 20%, 30%, 40%, 50%, 60%, 70%, 80%, 90%, and 100% of the AIBL dataset were used. The results show high prediction accuracy even for a training dataset with ten times smaller sample size compared to the size of the joining data.

The accuracy of the imputation method was very stable for different sizes of test and training data with the MAE only varying between 1.62 and 1.73 for LMII and between 1.44 and 1.58 for MMSE (Figs. 1 and 2). Further, accurate results were still obtained when the training (measured data) set was only 10% of the size of the test dataset (missing data) with MAEs of 1.73 and 2.52 for LMII and MMSE, respectively. The correlation between the actual and predicted values for the estimated LMII and MMSE scores showed similar accuracy for the different sizes of test and training datasets, varying between 0.91 and 0.92 for LMII and between 0.84 and 0.87 for MMSE (Figs. 3 and 4).

### Evaluation of imputed unmeasured data

**Figure 5:**
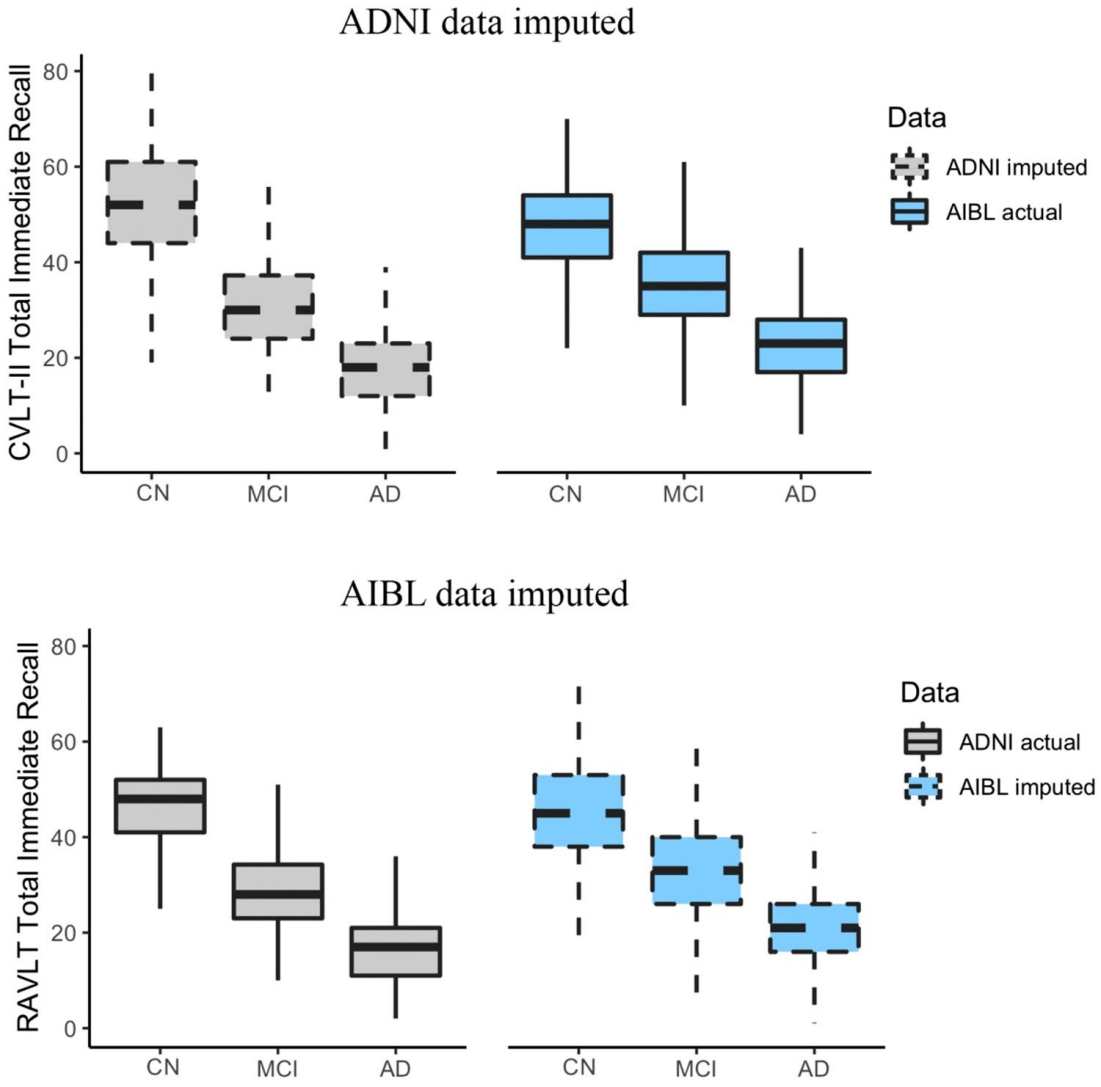
ADNI data imputed: distribution of the actual AIBL CVLT-II Total Immediate Recall scores and the imputed ADNI CVLT-II Total Immediate Recall scores for each clinical classification. AIBL data imputed: distribution of the actual ADNI RAVLT Total Immediate Recall score and the imputed AIBL RAVLT Total Immediate Recall score for each clinical classification.

A similarly high level of significant discrimination (p < 0.001) and large effect sizes (d > 1) between clinical classification was observed for the actual and the imputed scores of the CVLT-II and RAVLT (Fig. 5). The variance of the imputed variable in each group were comparable to the observed dataset (Fig. 5). The p-values and effect sizes are reported in Supplementary Tables S2 and S3.

### Data harmonisation utility case study

**Figure 6:**
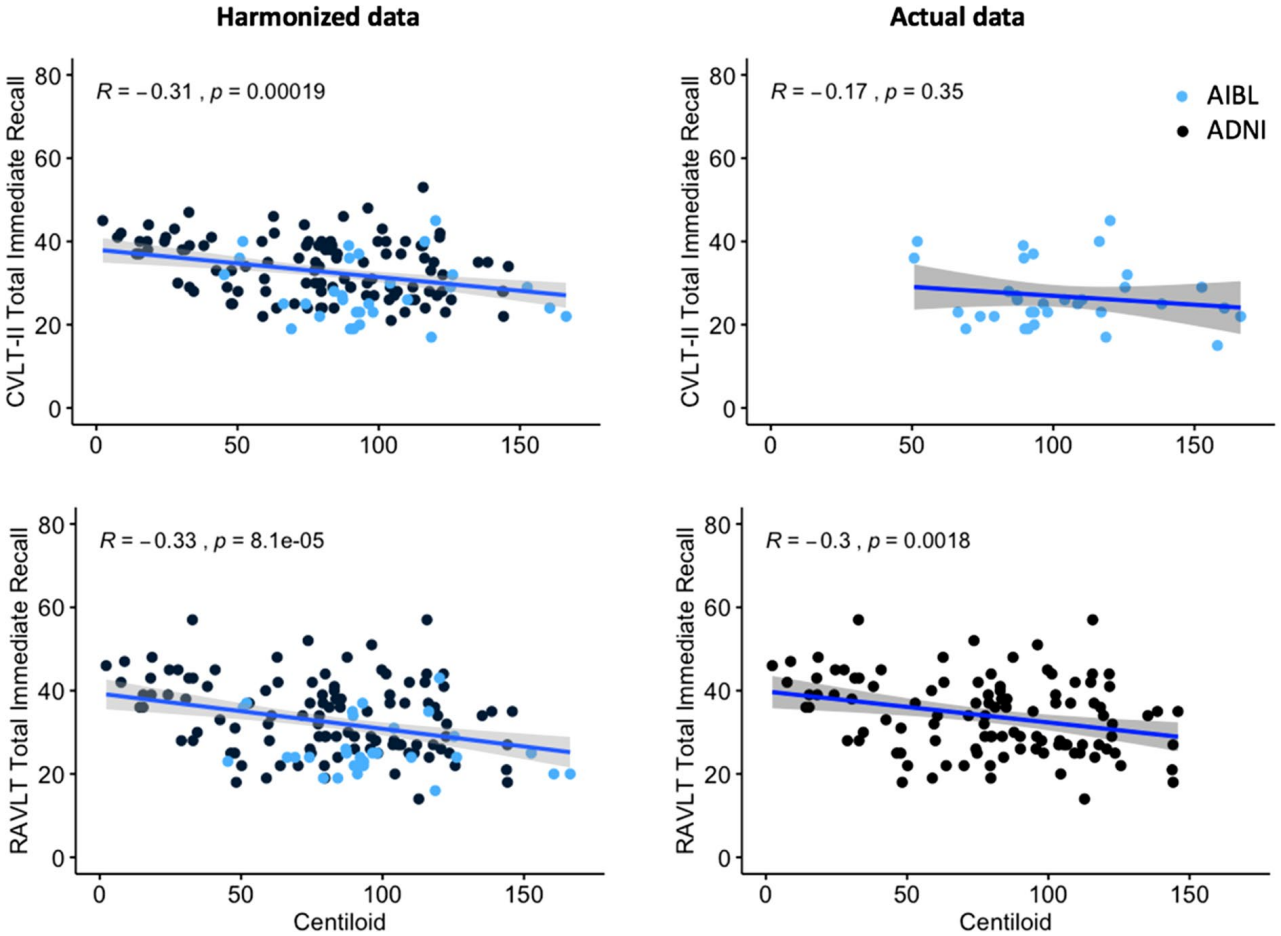
The association between Aβ level and memory performance measured with CVLT-II and RAVLT total immediate recall memory scores. Harmonized data include AIBL and ADNI data, presented in blue and black, respectively.

For the harmonized data, a significant negative correlation between CVLT-II scores and Aβ level in MCI who are *APOE*-ε4 homozygotes was observed (r = − 0.32, p < 0.001). No significant correlation was observed in actual AIBL data with the smaller sample size. A significant negative correlation was observed between RAVLT Total Immediate Recall scores and Aβ level in both the harmonized and actual ADNI data (r = − 0.32, p < 0.001; r = − 0.30, p < 0.001), refer to Fig. 6.

## Discussion

In this study, we have adapted a machine learning method, MissForest, for the harmonization of cognitive test scores. The method capitalises on the underlying structure and relationships of the datasets provided. Data across two separate datasets were used to impute data for a test not measured in one of the datasets to align it to a test measured in the other dataset. Results indicated that the method was successful at imputing data across datasets that could be combined to provide an overall harmonised dataset.

Treating unmeasured test scores as missing values and imputing missing scores using values from another dataset had comparable accuracy when imputing missing data within the same dataset, although accuracy was slightly higher when imputing using the same dataset. Further, increased accuracy was observed when AIBL was set as the test dataset in comparison to when ADNI was set as the test dataset. This could be explained by ADNI having more variability in the data due to a multi-centre collection strategy in comparison to two collection centres run under a central umbrella for AIBL. This may result in a lower ability to extract the underlying structure and relationships used for driving the imputation method in ADNI. Interestingly, it was shown that the range of accuracy does not change with different sample sizes for the test datasets, however using a bigger training dataset reflects a slightly improved performance. This is a promising result showing that even small datasets can be harmonized with larger publicly available datasets such as AIBL and ADNI. Further, it should be noted that these accurate results were obtained even though there were significant demographic differences between the AIBL and ADNI datasets, again suggesting that datasets with varying testing protocols can be effectively harmonised using this strategy.

Application of the method to actual missing data was able to show that imputed missing test scores (RAVLT in AIBL and CVLT-II in ADNI) and measured test data hold similar significant discrimination between clinical classifications. Note that clinical classifications of the subjects were not included in the test or training datasets. The case study also showed that in a very nuanced analysis (of MCI *APOE*-ε4 homozygotes), a correlation between Aβ levels and CVLT-II scores, which was not significant for the AIBL study prior to harmonization, was significant for the joined dataset after harmonisation. This result agrees with previous studies showing an association between cognitive performance and Aβ levels in MCI individuals^41–44^.

### Strengths of the study

One important strength of the proposed method is the capability of random forest based approaches to handle high dimensional mixed-type data^28^. Given that data from studies researching Alzheimer’s disease include continuous (e.g. age), discrete (e.g. MMSE) and categorical (e.g. gender) random variables, such a capability is essential for a harmonization method in this field. Secondly, the proposed method includes the rate of change for each of the clinical and cognitive tests as an input to the MissForest imputation model. This is important for health care data (e.g. Alzheimer’s data) to capture the temporal patient characteristics inherent in longitudinal measurements for each individual. Another crucial strength of the proposed procedure is that, unlike linear models such as multiple imputation by chained equations (MICE^45^), random forest is a non-parametric method and can handle nonlinearity within the data.

Further, in contrast to latent variable approaches^21,24^ that also use underlying information across datasets^26,27,46^, an additional ‘anchor’ dataset(s) that includes both of the unmeasured tests is not required for the method presented here.

### Limitations and future directions

The main limitation of the proposed approach is the need for a relatively rich dataset that includes both demographics and cognitive scores meaning that studies would need comparable data across these domains to undertake the harmonisation strategy. However, most cohort studies in Alzheimer’s disease research have both demographics and thorough neuropsychological test batteries.

There are two opportunities for improving the proposed method. Firstly, MissForest treats all data as individual data points and does not exploit the inherent structure of longitudinal data within subjects. In the future, extensions to MissForest (e.g. using mixed-effects random forest^47^ for missing data imputation) could be considered to provide improved imputation estimations as well as prognostic estimations into the future. Secondly, future work should consider the challenges presented by harmonizing more than two datasets, for example the number of tests to be imputed will exponentially increase with an increasing number of datasets and it will be necessary to ensure the method is robust in such scenarios.

## Summary

In summary, our results suggest using MissForest for data imputation can provide a practical solution for data harmonization across Alzheimer’s disease cohort studies, specifically AIBL and ADNI. Such harmonized datasets provide larger sample sizes and increased study power, in turn allowing more nuanced analyses to be undertaken and leading to improvements in the generalisability of findings.

## Supplementary Information

Supplementary Information.
