# Supplementary material for: Using imputation to provide harmonized longitudinal measures of cognition across AIBL and ADNI
Source: Sci Rep. 2021 Dec 10;11:23788. doi: 10.1038/s41598-021-02827-6 (PMC8664816; doi:10.1038/s41598-021-02827-6)
Supplement: Supplementary file 1 — Supplementary Information. [file 41598_2021_2827_MOESM1_ESM.pdf]

Using imputation to provide harmonized longitudinal measures of cognition across AIBL and ADNI

SUPPLEMENTARY MATERIALS

Rosita Shishegar[1,2*], Timothy Cox[1], David Rolls[1], Pierrick Bourgeat[1], Vincent Doré[1,3], Fiona Lamb[3], Joanne Robertson[4], Simon M. Laws[5,6,7], Tenielle Porter[5,6,7], Jurgen Fripp[1], Duygu Tosun[8], Paul Maruff[9], Greg Savage[10], Christopher C. Rowe[3,11], Colin L. Masters[4], Michael W. Weiner[8], Victor L. Villemagne[3,12], Samantha C. Burnham[1] for the Alzheimer's Disease Neuroimaging Initiative and the AIBL Study. *Correspondence to rosita.shishegar@csiro.au

1.The Australian e-Health Research Centre, CSIRO, Melbourne, Australia; 2.School of Psychological Sciences and Turner Institute for Brain and Mental Health, Monash University, Melbourne, Australia, 3.Department of Molecular Imaging & Therapy, Austin Health, Heidelberg, VIC, Australia; 4.Florey Institute of Neuroscience and Mental Health, The University of Melbourne, Parkville, VIC, Australia; 5. Centre for Precision Health, Edith Cowan University, Joondalup, Western Australia, Australia; 6.Collaborative Genomics and Translation Group, School of Medical and Health Sciences, Edith Cowan University, Joondalup, Western Australia, Australia; 7.School of Pharmacy and Biomedical Sciences, Faculty of Health Sciences, Curtin Health Innovation Research Institute, Curtin University, Bentley, Western Australia, Australia 8. Department of Radiology and Biomedical Imaging, University of California-San Francisco, San Francisco, CA, USA; 9.Cogstate Ltd, Melbourne, VIC, Australia; 10.Department of Psychology, Macquarie University, Sydney, NSW, Australia; 11. Department of Medicine, The University of Melbourne, Parkville, VIC 3052, Australia; 12. Department of Psychiatry, University of Pittsburgh School of Medicine, Pittsburgh, Pennsylvania, USA.

The Australian Imaging, Biomarkers and Lifestyle (AIBL) Study of Ageing

The AIBL study is a prospective longitudinal study of aging. The method for recruitment and enrolment have been described in detail elsewhere [1]. Briefly, individuals classified clinically with mild cognitive impairment (MCI) or dementia of the Alzheimer's type (AD) were recruited from primary-care physicians or tertiary Memory Disorders Clinics at two study centres in Melbourne, Victoria and Perth, Western Australia. Cognitively normal (CN) older adults were recruited through advertisement or from spouses of participants in the study, at the

same centres. 1112 eligible volunteers, who were aged 60 years or older and fluent in English were recruited by the AIBL study, between Nov 3, 2006, and Oct 30, 2008. A second cohort of 86 patients with AD, 124 MCI and 389 NC were recruited by AIBL between March 30, 2011, and June 29, 2015. The dataset contains data from neuroimaging, biomarkers, lifestyle, clinical information, and neuropsychological assessments. The follow-up data was collected every 18 months (18, 36, 54, 72 and 90 months) [1]. Please note that All assessments for all participant was not completed [1]. Written informed consent was obtained from all participants. Approval for the study was obtained from the human research ethics committees of Austin Health, St Vincent's Hospital, Edith Cowan University, and Hollywood Private Hospital (for more details, see [1], https://aibl.csiro.au).

All AIBL participants underwent extensive neuropsychological testing as described previously 38. Tests covering the main domains of cognition affected by AD and other dementias make up the AIBL clinical and neuropsychological battery. The full neuropsychological battery consisted of clinical measures: Clinical Dementia Rating (CDR), and Mini-Mental State Examination (MMSE) 39, and cognitive tests: California Verbal Learning Test – Second edition (CVLT-II) 20, Logical Memory (LM) I and II (WMS-III; Story A only), D-KEFS verbal fluency 40, 30-item Boston Naming Test (BNT) 41, the Stroop task (Victoria version) 20, the Rey Complex Figure Test (RCFT) 42, Digit Span and Digit Symbol-Coding subtests of the Wechsler Adult Intelligence Scale – Third edition (WAIS–III) 43, the Wechsler Test of Adult Reading 44, and Clock Drawing Test 45. Based on the inclusion criteria explaind in the next section the final dataset included 1805 AIBL participants (CN=1180, MCI=297 and AD=328), aged 72.42±7.64 years with 777 males at the baseline, and 2122 ADNI participants (CN=791, MCI=962, AD=369), aged 73.32±7.21 years with 1129 males at the baseline.

Alzheimer's Disease Neuroimaging Initiative (ADNI)

ADNI is a multi-centre longitudinal neuroimaging study, launched by the National Institute on Aging, the National Institute of Biomedical Imaging and Bioengineering, the Food and Drug Administration, private pharmaceutical companies and non-profit organizations in 2004. The dataset includes data from neuroimaging biomarkers, clinical information, and neuropsychological assessments, as previously described [2,3] (for more details, see http://www.adni-info.org/index). Subjects were recruited from 57 sites across the United States and Canada. initial ADNI recruitment, ADNI 1, included 200 CN subjects, 400 MCI subjects and 200 subjects with early AD. Then, ADNI GO, launched in 2009 included 200 subjects identified as having early mild cognitive impairment (EMCI). In 2011, ADNI 2 recruited 150 CN, 100 EMCI participants, 150 late mild cognitive impairment (LMCI) participants and 150 AD participants. In 2016, ADNI 3 was launched to recruit an additional 1200 volunteers. The ADNI participants were prospectively followed, with follow-up time points at three months, six months, then every six months until up to 156 months. A committee on human research at each participating institution approved the study protocol, and all participants gave their informed consent.

The ADNI participants underwent a battery of neuropsychological assessment at each study visit. The full ADNI neuropsychological battery includes the clinical measures of ADAS-Cog [4], Clinical Dementia Rating (CDR), and MMSE [5], and neuropsychological tests that assesses different cognitive domains, e.g. Rey Auditory Verbal Learning Test (RAVLT) [6], Story A from the Logical Memory Test [7], the Boston Naming Test (BNT) [8], animal and vegetable Category Fluency Test [9], Trail Making Test [10], Digit Span Test [7,11] and Digit Symbol Substitution Test [7] from the Wechsler Adult Intelligence Scale-Revised (WAIS-R), and the Clock Drawing Test [12].

Assessment of *APOE* genotype

In AIBL, 5 mL aliquots of blood were taken in ethylenediaminetetraacetic acid (EDTA)-containing vacutainer tubes from participants. Genomic DNA was extracted from whole blood using QIAamp DNA Blood Maxi Kits (Qiagen, Hilden, Germany) following the manufacturer's protocol. TaqMan® genotyping assays (Life Technologies) for rs7412 (Assay ID: C____904973_10) and rs429358 (Assay ID: C___3084793_20) were used to determine *APOE* genotype which is defined by the *APOE* ε2, ε3, and ε4 alleles. TaqMan® genotyping assays were performed on a QuantStudio 12K Flex™ Real-Time-PCR systems (Applied Biosystems, Foster City, CA) using the TaqMan® GTXpress™ Master Mix (Life Technologies) methodology as per manufacturer instructions [13]. In ADNI, 3 mL aliquots of blood were taken in EDTA-containing vacutainer tubes from participants. Genomic DNA was extracted at Cogenics (now Beckman Coulter Genomics) using the QIAamp DNA Blood Maxi Kit (Qiagen, Valencia, CA) following the manufacturer's protocol. The rs429358 and rs7412 SNPs were evaluated by polymerase chain reaction amplification, followed by HhaI restriction enzyme digestion, resolution on 4% Metaphor Gel, and visualization by ethidium bromide staining [14,15]. *APOE* carrier status was defined by the presence (1 or 2 copies) or absence (0 copies) of the *APOE*-ε4 allele.

Table S1. List of the included Alzheimer's Disease Assessment Scales and cognitive test scores, alongside their variable name in ADNI and AIBL datasets.

		**AIBL dataset**		**ADNI dataset**		**Joined dataset**	
	**Description**	**Variable name**	**Missing** [N (%)]	**Variable name**	**Missing** [N (%)]	**Variable name**	**Missing** [N (%)]

**The Boston Naming Test (BNT)**	Number of spontaneously given correct responses: Total correct without a cue	Neuropsych. BNT - No Cue (Australian RAW)	687 (10.9%)	BNTSPONT	1040 (10.5%)	BNT - No Cue	1727 (10.7%)
	Total correct with a semantic cue	Neuropsych. BNT - Stimulus Cued Score (Australian)	1032 (16.4%)	BNTCSTIM	1048 (10.6%)	BNT - Stimulus Cued Score	2080 (12.9%)
	Number of correct responses following a phonemic cue	Neuropsych. BNT - Phonemic Cued Score (Australian)	862 (13.7%)	BNTCPHON	1065 (10.8%)	BNT - Phonemic Cued Score	1927 (11.9%)
**Logical Memory Test**	Logical Memory - Immediate Recall Total Number of Story Units Recalled	Neuropsych. Recall RAW (LM1)	624 (9.9%)	LIMMTOTAL	4145 (42.0%)	LM1	4769 (29.5%)
	Logical Memory - Delayed Recall Total Number of Story Units Recalled	Neuropsych. Recall RAW (LMII)	676 (10.7%)	LDELTOTAL	2057 (20.8%)	LMII	2733 (16.9%)
**RAVLT: Rey Auditory Verbal Learning Test**	RAVLT Total immediate recall (sum of 5 trials)	-	-	RAVLT_immediate	149 (1.5%)	RAVLT Total immediate recall	6439 (39.8%)
	RAVLT Learning (trial 5 - trial 1)	-	-	RAVLT_learning	345 (3.5%)	RAVLT Learning	6635 (41.0%)
	RAVLT Forgotten (trial 5 - delayed)	-	-	RAVLT_forgetting	305 (3.1%)	RAVLT Forgotten	6595 (40.8%)

	RAVLT Percent forgotten	-	-	RAVLT_perc_forgetting	247 (2.5%)	RAVLT Percent forgotten	6537 (40.4%)
CVLT-II: California Verbal Learning Test-Second edition	CVLT-II Total immediate recall	Neuropsych. List A 1-5 RAW	788 (12.5%)	-	-	CVLT-II Total immediate recall	10663 (66.0%)
	CVLT-II free recall after short delay	Neuropsych. List A T6 Retention (RAW)	802 (12.8%)	-	-	CVLT-II Short Delay Free Recall	10677 (66.1%)
	CVLT-II free recall after long delay	Neuropsych. List A Delayed Recall (RAW)	828 (13.2%)	-	-	CVLT-II Long Delay Free Recall	10703 (66.2%)
	CVLT-II recognition after long delay	Neuropsych. List A Recognition (RAW)	943 (15.0%)	-	-	CVLT-II Long Delay Recognition - Hits	10818 (66.9%)
	CVLT-II False Positive recognition after long delay	Neuropsych. List A False Positives (RAW)	944 (15.0%)	-	-	CVLT-II Long Delay Recognition - False Positives	10819 (66.9%)
Clinical Dementia Rating Scale	Clinical Dementia Rating Scale (CDR) Sum of boxes	Neuropsych. CDR Sum of Boxes	389 (6.2%)	CDRSB	111 (1.1%)	CDR	500 (3.1%)
Mini-Mental State Examination	Mini-Mental State Examination (MMSE) total score	Neuropsych. MMSE	386 (6.1%)	MMSE	39 (0.4%)	MMSE	425 (2.6%)

Table S2. Between sub-group differences calculated for AIBL CVLT-II Total Immediate Recall scores compared with the imputed ADNI CVLT-II Total Immediate Recall scores Recall score.

**CVLT-II Total Immediate Recall score**	**Statistic (df)**	***p*-value**	**Effect size *d***
CN vs MCI	t(140.3)=18.9	<.001	1.9
MCI vs AD	t(642)=19.6	<.001	1.5
**RAVLT Total Immediate Recall scores**	**Statistic (df)**	***p*-value**	**Effect size *d***
CN vs MCI	t(115.2)=16.0	<.001	1.8
MCI vs AD	t(626.1)=19.1	<.001	1.5

Table S3. Between sub-group differences calculated for ADNI RAVLT Total Immediate Recall score compared with the imputed AIBL RAVLT Total Immediate Recall score.

**CVLT-II Total Immediate Recall score**	**Statistic (df)**	***p*-value**	**Effect size *d***
CN vs MCI	t(631.4)=12.8	<.001	1.0
MCI vs AD	t(580.8)=11.2	<.001	1.4
**RAVLT Total Immediate Recall scores**	**Statistic (df)**	***p*-value**	**Effect size *d***
CN vs MCI	t(643.3)=12.6	<.001	1.0
MCI vs AD	t(573.9)=12.1	<.001	1.5

Data harmonization application: sensitivity analyses on CVLT-II and RAVLT

The sensitivity of imputed CVLT-II and RAVLT scores was examined using simulated missing values of AIBL and ADNI, respectively. For CVLT-II Total Immediate Recall scores, the AIBL sample was split randomly into a 10% simulated missing data and 90% observed data set. Then the method imputed all missing values including simulate missing CVLT-II Total Immediate Recall scores in AIBL and unmeasured CVLT-II scores in AD. The performance of the data imputation was examined on the AIBL simulated missing values using MAE and

the Pearson correlations between the estimated and the actual scores. Similarly, for RAVLT scores, the ADNI sample was split randomly into a 10% simulated missing data and 90% observed data set. Then the method imputed all missing values including simulate missing RAVLT Total Immediate Recall scores in ADNI as well as unmeasured RAVLT scores in AIBL. The performance of the data imputation was examined on the ADNI simulate missing values using MAE and the Pearson correlations between the estimated and the actual scores.

Results: sensitivity analyses on CVLT-II and RAVLT

The mean absolute errors (MAE; mean±standard error) of the sensitivity analysis indicated that the imputed AIBL CVLT-II Total Immediate Recall (range 0-80) and ADNI RAVLT Total Immediate Recall (range 0-75) scores estimated using the observed part of the AIBL and ADNI datasets were 5.15±.04 and 4.73±.02, respectively. The correlation (mean±standard error) between the imputed values for AIBL CVLT-II Total Immediate Recall and ADNI RAVLT Total Immediate Recall scores estimated using the observed part of the datasets were .92±.00 and .89±.00, respectively.

REFERENCES

1 Ellis, K. A. *et al.* The Australian Imaging, Biomarkers and Lifestyle (AIBL) study of aging: methodology and baseline characteristics of 1112 individuals recruited for a longitudinal study of Alzheimer's disease. *International psychogeriatrics* **21**, 672-687 (2009).

2 Jack Jr, C. R. *et al.* The Alzheimer's disease neuroimaging initiative (ADNI): MRI methods. *Journal of Magnetic Resonance Imaging: An Official Journal of the International Society for Magnetic Resonance in Medicine* **27**, 685-691 (2008).

3 Petersen, R. C. *et al.* Alzheimer's disease neuroimaging initiative (ADNI): clinical characterization. *Neurology* **74**, 201-209 (2010).

4 Rosen, W. G., Mohs, R. C. & Davis, K. L. A new rating scale for Alzheimer's disease. *The American journal of psychiatry* (1984).

5 Snyder, P. J. *et al.* Assessing cognition and function in Alzheimer's disease clinical trials: do we have the right tools? *Alzheimer's & Dementia* **10**, 853-860 (2014).

6 Rey, A. & en Psychologie, E. C. (Paris, 1964).

7 Wechsler, D. Wechsler memory scale-revised. *Psychological Corporation* (1987).

8 Kaplan, E., Goodglass, H. & Weintraub, S. The Boston Naming Test. Lea & Febiger. *Philadelphia, PA* (1983).

9 Butters, N., Granholm, E., Salmon, D. P., Grant, I. & Wolfe, J. Episodic and semantic memory: A comparison of amnesic and demented patients. *Journal of clinical and Experimental Neuropsychology* **9**, 479-497 (1987).

10 Reitan, R. M. Validity of the Trail Making Test as an indicator of organic brain damage. *Perceptual and motor skills* **8**, 271-276 (1958).

11 Stekhoven, D. J. Using the missForest package. *R package*, 1-11 (2011).

12 Goodglass, H. & Kaplan, E. The Assessment of Aphasia and Related Disorders, 2nd edn Lea & Febiger: Philadelphia. *Dictionary of Biological Psychology* **230** (1983).

13 Porter, T. *et al.* A polygenic risk score derived from episodic memory weighted genetic variants is associated with cognitive decline in preclinical Alzheimer's disease. *Frontiers in aging neuroscience* **10**, 423 (2018).

14 Potkin, S. G. *et al.* Hippocampal atrophy as a quantitative trait in a genome-wide association study identifying novel susceptibility genes for Alzheimer's disease. *PloS one* **4**, e6501 (2009).

15 Saykin, A. J. *et al.* Alzheimer's Disease Neuroimaging Initiative biomarkers as quantitative phenotypes: genetics core aims, progress, and plans. *Alzheimer's & dementia: the journal of the Alzheimer's Association* **6**, 265-273 (2010).
